# TTC6-Mediated Stabilization of the Flagellum Annulus Ensures the Rapid and Directed Motion of Sperm

**DOI:** 10.3390/cells12162091

**Published:** 2023-08-18

**Authors:** Ziqi Wang, Kailun Fang, Yanling Wan, Yingying Yin, Mengjing Li, Ke Xu, Tongtong Li, Yongzhi Cao, Yue Lv, Gang Lu, Hongbin Liu, Tao Huang

**Affiliations:** 1 Center for Reproductive Medicine, Shandong University, Jinan 250012, China; wangziqi1110@126.com (Z.W.); wyl9208@163.com (Y.W.); 18865381921@163.com (Y.Y.); lmjsduer@163.com (M.L.); 202020745@mail.sdu.edu.cn (K.X.); litongtong199410@163.com (T.L.); yz_cao@163.com (Y.C.); lugang@cuhk.edu.hk (G.L.); hongbin_sduivf@aliyun.com (H.L.); 2 Key Laboratory of Reproductive Endocrinology of Ministry of Education, Shandong University, Jinan 250012, China; 3 Shandong Provincial Clinical Medicine Research Center for Reproductive Health, Jinan 250012, China; 4 Shandong Technology Innovation Center for Reproductive Health, Jinan 250012, China; 5 Institute of Neuroscience, State Key Laboratory of Neuroscience, CAS Center for Excellence in Brain Science and Intelligence Technology, Chinese Academy of Sciences, Shanghai 200031, China; fangkailun@sibcb.ac.cn; 6 The Model Animal Research Centre, Shandong University, Jinan 250010, China; 7 CUHK-SDU Joint Laboratory on Reproductive Genetics, School of Biomedical Sciences, The Chinese University of Hong Kong, Hong Kong, China; lvyue0618@163.com; 8 Shandong Key Laboratory of Reproductive Medicine, Shandong First Medical University, Jinan 250012, China; 9 Research Unit of Gametogenesis and Health of ART-Offspring, Chinese Academy of Medical Sciences, Jinan 250012, China

**Keywords:** TTC6, subfertility, sperm, flagellum, annulus

## Abstract

Sperm motility and structural integrity are essential for successful fertilization in vivo, and any hindrance of the correct assembly of the axoneme and peri-axonemal structures in the sperm flagellum can lead to fertility problems. While there has been considerable advancement in studying diseases related to the flagellum, the underlying mechanisms that control sperm movement are not yet fully understood. In this study, we reveal that the tetratricopeptide repeat protein 6 (*Ttc6*) gene, expressed mainly in the testes, plays a crucial role in maintaining male fertility in mice. We further demonstrate that the knockout of *Ttc6* in mice results in decreased sperm motility and induces an abnormal circular swimming pattern, consequently leading to male subfertility. Morphological analysis showed an atypical hairpin-like appearance of the spermatozoa, and ultrastructural studies showed unsheathed flagella at the juncture between the midpiece and principal piece. Collectively, these findings suggest that TTC6 plays an essential role in maintaining the stability of the annulus region of the sperm flagellum, thus ensuring the swift and directed motion of sperm.

## 1. Introduction

Mammalian sperm, akin to various other vertebrate species, carry their haploid DNA within the head and employ a flagellum for movement [1,2]. This complex structure, seen across different eukaryotic cells, is composed of three distinct sections: the midpiece, the principal piece, and the end piece, with the axoneme spanning these segments [3]. Within the midpiece, one can find mitochondria, as well as outer dense fibers and a fibrous sheath that reaches to the principal piece, in contrast to the endpiece, which does not contain any peri-axonemal components. The axoneme, serving as the core structure of flagella and cilia, is constructed from microtubule doublets and a range of accessory elements including dynein motors, radial spokes, and the nexin-dynein regulatory complex [4]. This structure exhibits a 9 + 2 arrangement, which consists of two individual singlet microtubules (C1 and C2), encircled by a cylindrical formation containing nine sets of microtubule doublets. These central microtubules are linked by periodic bridges and are enveloped by the central pair projection, also referred to as the inner sheath [5,6]. Fine axoneme structures contribute to the cilium bending and axonemal integrity that are essential for the proper functioning of flagella and cilia [4].

The annulus is a ring structure in the sperm flagellum that is closely associated with sperm health and fertility [7]. A recent study on mammalian sperm has shed light on the proteins and processes involved with the annulus, and a group of proteins known as septins has been found to be localized to the annulus [8]. Research in mice has demonstrated that the annulus acts as a protein diffusion barrier and organizes the midpiece of the sperm tail, and the absence of the annulus has been linked to flagellum differentiation issues and to asthenozoospermia in humans. These studies have highlighted the essential role of the annulus in sperm health and motility and have suggested that a lack of septins or TAT1 at the annulus can be used as a diagnostic marker for certain types of male infertility [9,10,11,12].

Sperm motility is a crucial factor for successful fertilization and is generated by the controlled sliding of outer microtubule doublets facilitated by inner and outer dynein arms [13]. This interaction between dyneins and microtubules leads to flagellar bending and the generation of a bending force [14]. The activity of dyneins is additionally modulated by structures including the radial spokes and a central duo of microtubules [15]. To ensure high-speed swimming and to maintain its robustness against its own beating motion, the flagellum relies on multiple microtubule inner proteins, along with peri-axonemal components such as the outer dense fibers and the intricate fibrous sheath [16,17]. Within the flagellum, accessory elements such as the nexin-dynein regulatory complex, in conjunction with radial spokes, serve to reinforce the structure, thereby safeguarding against deformation [13]. The nature of the sperm cell’s swimming trajectory is dictated by the balance or imbalance in the flagellar beating. Specifically, a symmetrical beat pattern results in a linear motion, while any deviation from symmetry causes the sperm cell to follow a curved or potentially spiral course [18,19]; when flagellar defects occur, they can impede sperm motility and fertility. Despite several genes being linked to such defects, including *Tekt4* [20], *Tecte1* [21], *Cfap97d1* [22], and *Ttll3* and *Ttll8* [23], the mechanism of flagellar beat regulation remains largely unexplored.

The tetratricopeptide repeat protein family, including TTC21a, TTC26, TTC29, TTC30a, and TTC30b, plays a significant role in forming and operating the ciliar and flagellar structures that are pivotal for cell movement and signal transduction [24,25,26,27,28]. To date, only mutations in *TTC21a* and *TTC29* have been associated with multiple morphological abnormalities of the flagella, a condition that precipitates morphological abnormalities in sperm flagella and can result in male infertility [25,26]. Tetratricopeptide repeat protein 6 (TTC6) is another member of the tetratricopeptide repeat protein family and is considered a candidate gene for ciliopathies according to recent research into the morbid genome of ciliopathies. For example, patients with Meckel–Gruber syndrome carrying *TTC6* mutations display impaired ciliogenesis potential [29]. However, the specific functions of TTC6 in preserving sperm motility and the integrity of the flagellar structure remain poorly understood.

To elucidate the biological function of *Ttc6* in vivo, we generated a *Ttc6* knockout mouse model. Knockout of *Ttc6* resulted in diminished sperm motility and induced circular sperm swimming, ultimately disrupting fertilization and causing male subfertility. The morphological analysis of epididymal spermatozoa revealed a hairpin phenotype. Furthermore, ultrastructural studies showed that knockout of *Ttc6* led to unsheathed flagella at the juncture between the midpiece and the principal piece. These observations suggest that TTC6 serves to reinforce the annulus region, thus preserving the integrity of the sperm flagellum and ensuring the rapid and directed movement of sperm.

## 2. Method

### 2.1. Mice

The *Ttc6*-knockout mouse model was generated on a C57BL/6 background using CRISPR/Cas-mediated genome engineering (Cyagen Biosciences Inc., Guangzhou, China). Based on the *Ttc6* gene sequence (Ensembl: ENSMUSG00000046782), exon 2 to exon 32 was selected as target site and was deleted. For the production of knockout mice, Cas9 mRNA and gRNA, synthesized through in vitro transcription, were subsequently injected into fertilized eggs. The genotyping of the resultant mice was performed using PCR, followed by an examination through DNA sequencing analysis. In this study, RT-PCR experiments were also used to determine the *Ttc6* genotype. All the genotyping primers were as follows: for PCR and DNA sequencing, forward 5′-CAG CTA CGG GGC ATG GAG GTA C-3′ and 5′-CTG AAA TCC TAC ACT TGT GAG ACT GGA GC-3′ and reverse 5′-AAC ATA TTC AAC AGT ATC CAG CTT GAA AAG A-3′; for RT-PCR, forward 5′-AAC TCG TGC CAT CCA TCT CC-3′ and reverse 5′-GGG ATT TGC TTT CAC GGC TT-3′. All procedures involving animal care and experiments were examined and sanctioned by the Animal Use Committee at the School of Medicine, Shandong University. Compliance with all relevant institutional and national guidelines for the humane treatment and utilization of animals was strict. 

### 2.2. Histology, Immunostaining, Electron Microscopy, TUNEL Assay

For the purpose of histological analysis, sections of testes and epididymides were embedded in paraffin and cut to a thickness of 5 μm. These sections were subsequently stained utilizing Periodic acid-Schiff (PAS) and hematoxylin, as needed. The identification of stages within the seminiferous epithelium cycle and the various steps involved in spermatid development were conducted according to previously established methods.

For the analysis using transmission electron microscopy, sections of testes were initially fixed employing a solution of 2.5% phosphate-buffered glutaraldehyde. Following this step, the specimens underwent a dehydration process via a series of ethanol solutions with concentrations ranging from 50% to 100%. The samples were then infiltrated with pure acetone, embedded within Epon 812 resin, and sectioned using an ultramicrotome. Staining was achieved with a combination of uranyl acetate and lead citrate. Finally, the testes sections were examined and captured using a TECNAI-10 (Philips), Amsterdam, Holland transmission electron microscope, operating at an acceleration voltage of 80 kV. 

For the purpose of scanning electron microscopy analysis, pieces of the cauda epididymis were swiftly dissected and placed in 1 mL of PBS solution. Following the mincing of the tissue, a 10 µL aliquot of sperm fluid from each specimen was placed on individual coverslips. These were then fixed with 2.5% glutaraldehyde and subsequently postfixed with osmium tetroxide. A series of washes with water was performed, at least three times, followed by a sequential dehydration process through ethanol solutions of varying concentrations (50%, 70%, 95%, and 100%). The coverslips were then dried at a critical point of 55 °C using a Tousimis Autosamdri-810 Critical Point Dryer. Once dried, the specimens were mounted on stubs, sputter-coated with palladium, and finally examined and imaged using an FEI Quanta ESEM 200 scanning electron microscope. 

For the immunostaining procedure, sections of testicular tissue were initially fixed using 4% paraformaldehyde, followed by permeabilization with a 0.2% Triton X-100 solution in PBS. The sections were then exposed to primary antibodies specifically chosen for the process, which included mouse anti-acetylated α-tubulin (diluted 1:1000; Sigma Aldrich, St. Louis, MO, USA # T7451) and rabbit antiglycylation on β-tubulin (diluted 1:15,000; AdipoGen, Liestal, Switzerland # AG-25B-0034). These primary antibodies were subsequently detected using either Alexa Fluor 488- or 594-conjugated secondary antibodies at a dilution of 1:500 (Abcam, Cambridge, UK #ab150084 and #ab150113), with an incubation period of 1 h at ambient temperature. Following several washes with PBS, the slides were prepared with VECTASHIELD antifade mounting medium that included DAPI (Vector Laboratories, Burlingame, CA, USA #H-1200), and the resulting immunofluorescence (IF) images were promptly captured with the aid of either an LSM 780/710 microscope (Zeiss, Oberkochen, Germany) or an SP8 microscope (Leica, Wetzlar, Germany).

### 2.3. CASA 

Sperm samples were collected from both *Ttc6^+/+^* and *Ttc6^−/−^* mice, with a minimum of three mice utilized for each genotype. The motility of these sperm was subsequently analyzed using an Olympus B×51 microscope (Olympus, Tokyo, Japan) equipped with a 20× phase objective. Specifically, 10 μL aliquots of each sample were placed into 80 mm deep glass cell chambers (Hamilton Thorne, Beverly, MA, USA 80 mm 2X-CEl) and images were captured using a CCD camera. Utilizing the Animal Motility system (Hamilton Thorne, Beverly, MA, USA), computer-associated semen analysis was carried out on more than 200 spermatozoa from each sample. This part of the analysis was repeated a minimum of five times for consistency. 

### 2.4. Mouse Sperm Collection

The caudal epididymides from the *Ttc6^+/+^* and *Ttc6^−/−^* mice were carefully dissected. Spermatozoa were then extracted from the caudal epididymis and allowed to incubate in 1 mL of phosphate-buffered saline (PBS) at a temperature of 37 °C and under a 5% CO_2_ atmosphere for 30 min, to facilitate the sperm-counting experiments. 

### 2.5. Tissue Collection and Histological Analysis

Immediately following euthanasia, the testes and caudal epididymides were extracted from *Ttc6^+/+^* and *Ttc6^−/−^* mice. The specimens were rapidly fixed using 4% (*m*/*v*) paraformaldehyde (PFA; Solarbio, Beijing, China, P1110) and then preserved for a period not exceeding 24 h. Following this, the samples were dehydrated with 70% (*v*/*v*) ethanol and embedded in paraffin. Sections of 5 mm thickness were prepared, mounted onto glass slides, and subsequently stained with H&E for histological examination. In addition to this, Bouin’s fixatives (Polysciences, Warrington, PA, USA) were employed to fix testes specifically for PAS staining. Upon deparaffinization, slides were stained utilizing both PAS and H&E. This allowed for a detailed assessment of the various stages of the seminiferous epithelium cycle, as well as the progression of spermatid development. 

### 2.6. Flagellar Movement of Tethered Sperm Head

Flagellar waveform analysis via tethering sperm heads for planar beating was performed as described previously. Briefly, spermatozoa from the dissected cauda epididymis from adult *Ttc6^+/+^* and *Ttc6^−/−^* male were incubated for 15 min in 37 °C prewarmed PBS medium. Next, spermatozoa were plated on 35 mm gelatin-coated coverslips of a cell culture dish (Cellvis) for 15 min, unattached sperm were removed with a gentle pipette wash, and attached sperm cells were incubated in PBS medium. Flagellar movements of the tethered sperm were recorded at 37 °C for 3 s as a video at 60 fps. Utilizing a high-speed camera coupled with an inverted microscope, images were captured. Dark-field imagery was attained at a rate of 200 frames per second (fps) with the aid of an LED illumination source. Subsequent to capturing, the images were subjected to analytical processing. This included the application of a Gaussian blur, characterized by a sigma value of 0.5 pixels, and the utilization of the subtract background technique with a radius set at 5 pixels, all of which were executed within the ImageJ software (v1.4.3.67) framework. 

### 2.7. In Vivo Fertilization Assay

Wild-type female mice, within the age range of 4 to 6 weeks, were subjected to superovulation through intraperitoneal administration of five units of pregnant mare serum gonadotropin, succeeded by five units of human chorionic gonadotropin (hCG) administered 46–48 h subsequently. These females were then paired with male mice of both *Ttc6^+/+^* and *Ttc6^−/−^* genotypes. Following mating, oviducts bearing copulation plugs were carefully removed to recover either two-cell stage embryos or blastula-stage embryos at intervals of 45 and 96 h post-hCG injection, respectively. 

### 2.8. Sperm–Egg Binding Test

Wild-type ovum samples were processed with 0.01% (*w*/*v*) hyaluronidase (H-3757; Sigma) to exclude cumulus cells and then positioned within a 0.15 mL volume of HTF media, sheltered with mineral oil. A measured aliquot of capacitated cauda epididymal sperm (2–4 × 10^5^ sperm) was subsequently added. After a 3 h coincubation duration, the ova were gently washed with HTF solution to detach loosely adherent and unattached sperm cells.

### 2.9. In Vitro Fertilization Assay

Oocyte–cumulus complexes (OCCs) were extracted from superovulated female mice 14 h after hCG injection, and a fraction of these OCCs was subjected to 0.01% (*w*/*v*) hyaluronidase to acquire cumulus-free oocytes. Following this, either OCCs or the obtained cumulus-free oocytes were incubated with capacitated epididymal sperm within an environment of 37 °C and 5% CO_2_. After a 6 h incubation, the eggs were cleansed and transferred to KSOM culture medium, facilitating further developmental progression. 

### 2.10. Statistical Analysis

Data were statistically analyzed and are reported as mean values accompanied by the SEM. The majority of experimental procedures were performed with no less than three distinct samples, and each procedure was replicated a minimum of three times. Comparative analysis of the results across two experimental groups was carried out utilizing a two-tailed unpaired Student’s *t*-test. Significance levels for this test were categorized as follows: *p* < 0.05, *p* < 0.01, and *p* < 0.001, symbolized by asterisks (*), (**), and (***), respectively. A notation of “n.s.” was employed to signify statistical nonsignificance. 

## 3. Results

### 3.1. Ttc6 Is a Testis-Enriched Gene

**Figure 1:**
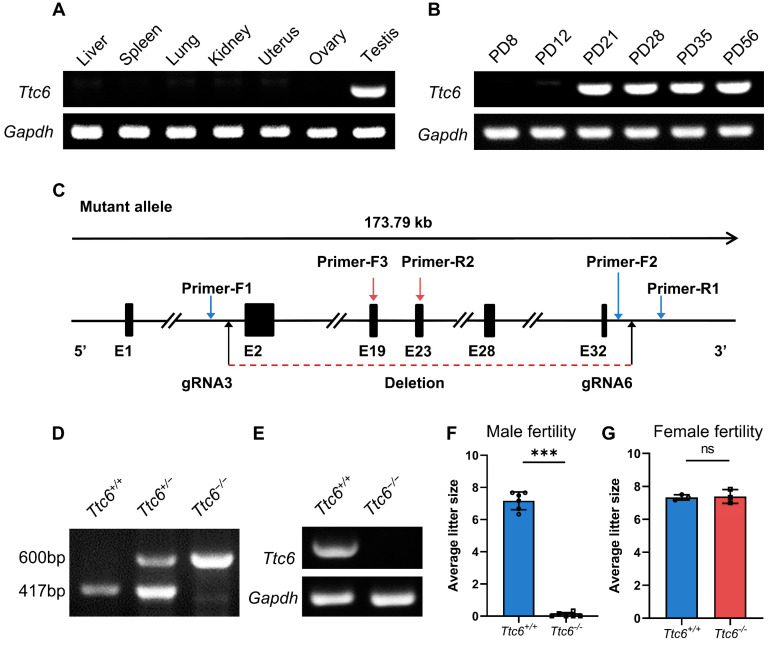
*Ttc6* knockout leads to male subfertility in mice. (**A**) Tissue expression profiles of *Ttc6* mRNA from various adult mouse tissues were measured with RT-PCR. *Gapdh* mRNA was amplified as the internal control. (**B**) The tissue expression profile of *Ttc6* mRNA from the different ages of postnatal testis as measured using RT-PCR. *Gapdh* mRNA was amplified as the internal control. (**C**) A schematic illustration of the knockout strategy for generating *Ttc6^−/−^* mice. gRNA3 and gRNA6 were designed to delete the region from exon 2 to exon 32. Primer sites utilized in the study are designated as follows: For (**D**), the WT and heterozygote were produced using Primer-F2 and Primer-R1, with a product of 417 bp, and the mutant allele was produced using Primer-F1 and Primer-R1 with a product of 600 bp. For Figure 1E, RT-PCR analysis was performed with Primer-F3 and Primer-R2 as the primer sites, with a product of 463bp. (**D**) Genotyping identification of *Ttc6*-knockout mice. (**E**) *Ttc6* mRNA was detected in the testes from wild-type mice but not *Ttc6^−/−^* mice. (**F**) The average litter size of *Ttc6^+/+^* and *Ttc6^−/−^* male mice at 3 months (*n* = 6 independent experiments). *Ttc6^−/−^* male mice were subfertile. Data are presented as the mean ± SD. *** *p* < 0.001. (**G**) All tested *Ttc6^−/−^* females were fertile. The average litter size of *Ttc6^+/+^* and *Ttc6^−/−^* female mice at 3 months (*n* = 4). ns, no statistical significance.

Utilizing RNA profiling data sourced from the Mouse ENCODE project and NCBI (https://www.ncbi.nlm.nih.gov/gene/70846, accessed on 1 July 2023), our analysis revealed that the expression of *Ttc6* mRNA transcripts is highly concentrated in the testes and bladder of mice. In contrast, the remaining mouse organs demonstrated only trace levels of these transcripts. To determine the specific expression profile of *Ttc6*, we performed a multitissue RT-PCR analysis in adult mice. Our findings indicated a high degree of *Ttc6* expression in the testis but negligible expression in other organs (Figure 1A). RT-PCR on postnatal testes at different ages showed that *Ttc6* was initially expressed from the postnatal day 21 and was then expressed continuously thereafter in adult mice (Figure 1B). This timeline corresponded with the stage of sperm condensation and flagellum formation, implying that TTC6 has an essential role in flagellation.

### 3.2. Ttc6-Knockout Male Mice Have Severe Fertility Defects

Situated on chromosome 12, the *Ttc6* gene spans 173.79 kb and is specific to the testis, commencing with an ATG start codon in exon 2 and terminating with a TGA stop codon in exon 32. To investigate the potential functions of *Ttc6* in the process of spermatogenesis, we engineered *Ttc6*-knockout mice through the application of the CRISPR/Cas9 genome editing technology, supplied by Cyagen Biosciences, Santa Clara, CA, USA. Exons 2 through 32 were chosen as the target sites for this procedure (Figure 1C). Genotyping of the founder animals was conducted using genomic DNA sequencing, and validation was achieved through polymerase chain reaction. In *Ttc6^+/+^* male mice, the *Ttc6* locus was found to be 417 bp, contrasting the 600 bp size of the locus in *Ttc6^−/−^* male mice (Figure 1D). No mRNA was detected by RT-PCR in *Ttc6*-knockout testes (Figure 1E). 

We did not observe any noticeable abnormalities in the development, behavior, or survival rates of the knockout mice. To assess the fertility of both male and female *Ttc6^−/−^* mice, we bred not only adult knockout male mice but also female mice with wild-type counterparts for a period of 3 months. *Ttc6^+/+^* males that sired a normal number of offspring (Figure 1F and Figure S1A) served as control subjects throughout the research. The control *Ttc6^+/+^* males averaged 7.17 ± 0.23 (standard error of the mean (SEM), *n* = 6) offspring per litter, while *Ttc6^−/−^* males sired a considerably lower average of 0.08 ± 0.05 (SEM, *n* = 6, Figure 1F and Figure S1A). Remarkably, two-thirds (66%) of the *Ttc6^−/−^* males failed to sire any offspring at all. In contrast to the males, female fertility appeared to be unaffected (Figure 1G). The subfertility observed in *Ttc6^−/−^* mice does not appear to be a result of hormonal alterations because testosterone levels were found to be consistent (Figure S1B). Therefore, we inferred that the *Ttc6* gene is required for maintaining normal male fertility in mice.

### 3.3. Ttc6^−/−^ Males Are Subfertile but Have Normal Spermatogenesis

**Figure 2:**
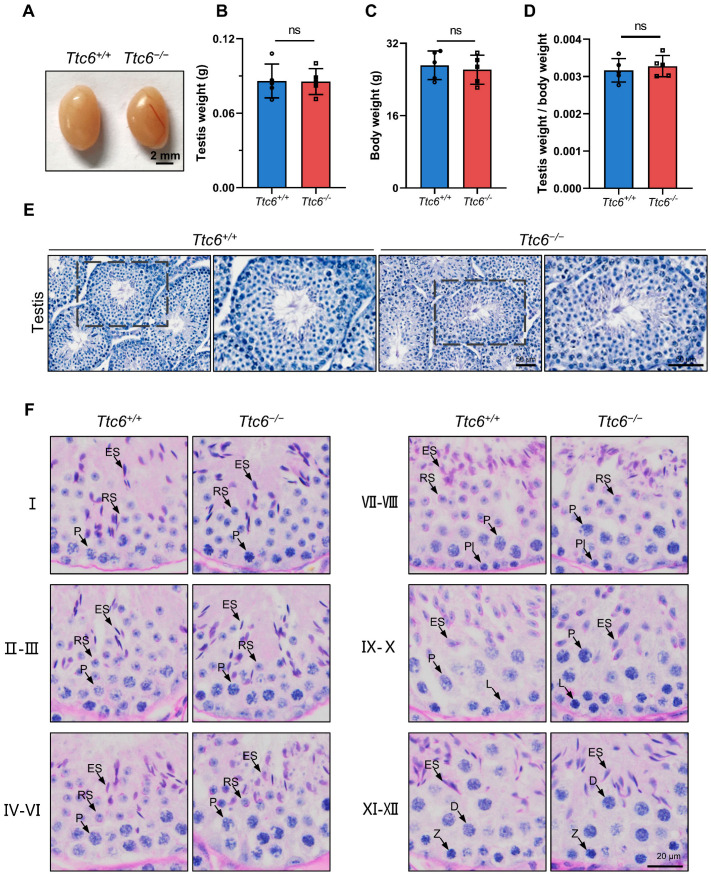
Morphological and microscopic images of testes in *Ttc6^−/−^* mice. (**A**) The size of the testis was not obviously different between *Ttc6^+/+^* and *Ttc6^−/−^* mice. Scale bar, 2 mm. (**B**) The testis weights of *Ttc6^+/+^* and *Ttc6^−/−^* mice. Data are presented as the mean ± SEM, *n* = 5. n.s., no statistical significance. (**C**) The body weights of *Ttc6^+/+^* and *Ttc6^−/−^* mice. Data are presented as the mean ± SEM, *n* = 5. n.s., no statistical significance. (**D**) The ratio of testis weight to body weight in *Ttc6^+/+^* and *Ttc6^−/−^* mice. Data are presented as the mean ± SEM, *n* = 5. n.s., no statistical significance. (**E**) Hematoxylin staining indicating that the histomorphology of *Ttc6^−/−^* seminiferous tubules was similar to that of *Ttc6^+/+^* seminiferous tubules. Scale bar, 50 μm. (**F**) PAS–hematoxylin staining analysis of the testis seminiferous tubule cross-sections in *Ttc6^+/+^* and *Ttc6^−/−^* mice. Arrows highlight germ cells at various stages of spermatogenesis. Pl, preleptotene; L, leptotene; Z, zygotene; P, pachytene; D, diplotene; RS, round spermatids; ES, elongating spermatids. Scale bar, 20 μm.

The morphology and motility of sperm are essential factors that predict the success rates of oocyte fertilization and subsequent pregnancy. Any irregularities within these parameters can be a substantial contributing factor to subfertility. To further investigate the cause of the subfertility in male knockout mice, we initially scrutinized the structure of the adult *Ttc6^−/−^* testis at both the macroscopic and microscopic levels. No significant variances were observed in the size of the testis (Figure 2A), the weight of the testis (Figure 2B), the overall body weight of the mouse (Figure 2C), or the proportion of testicular weight relative to the total body mass (Figure 2D) when comparing *Ttc6^−/−^* and *Ttc6^+/+^* male mice. Hematoxylin staining indicated that the seminiferous tubule structure in *Ttc6^−/−^* testes remained normal (Figure 2E), and periodic acid-Schiff (PAS)–hematoxylin staining revealed that spermatogenesis proceeded normally and that the acrosomal and nuclear morphology of spermatids in *Ttc6^−/−^* mice was intact (Figure 2F and Figure S2A). These findings support the hypothesis that disruption of *Ttc6* has little effect on acrosome biogenesis.

**Figure 3:**
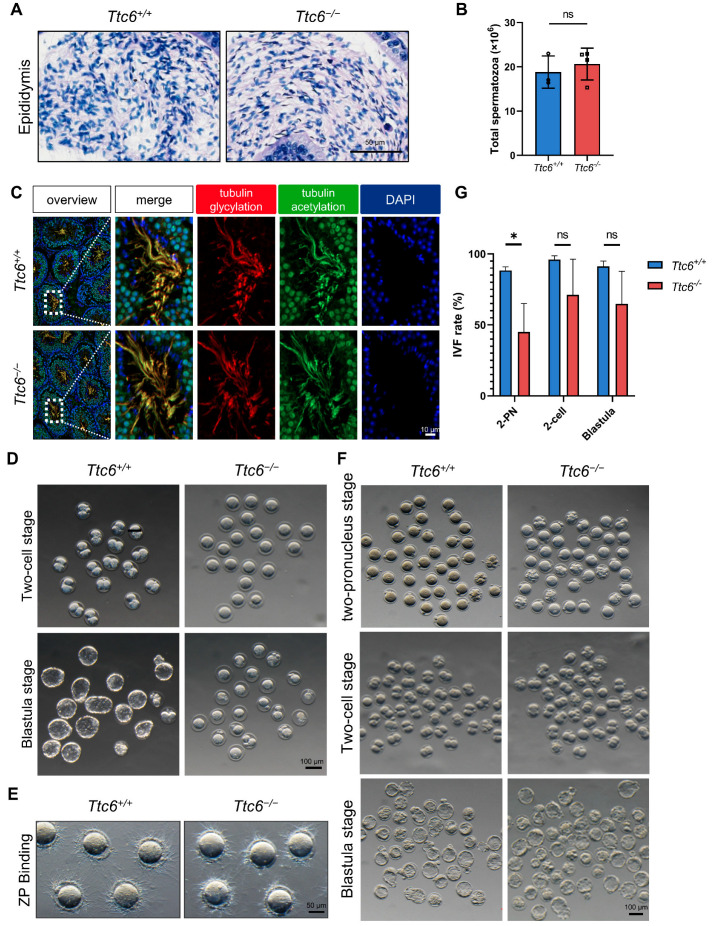
Fertilization capacity of *Ttc6^−/−^* sperm in vivo and in vitro. (**A**) Hematoxylin staining of the caudal epididymis of *Ttc6^+/+^* and *Ttc6^−/−^* mice. Scale bar, 50 μm. (**B**) Sperm counts in the caudal epididymis of *Ttc6^−/−^* mice (*n* = 3) did not differ from those of *Ttc6^+/+^* mice (*n* = 4). Data are presented as the mean ± SEM. Two-tailed Student’s *t*-test; ns: no significance. (**C**) Immunofluorescence staining of mouse tubulin acetylation (green), glycylation (red), and DAPI in testes of 12-week-old *Ttc6^+/+^* and *Ttc6^−/−^* mice. Scale bars, 10 µm. (**D**) Fertilized oocytes collected from superovulated wild-type females plugged by *Ttc6^+/+^* and *Ttc6^−/−^* males. Morphology of two-cell–stage and blastula-stage embryos. Scale bar, 100 μm. (**E**) Sperm-zona pellucida binding assay with *Ttc6^+/+^* and *Ttc6^−/−^* mice spermatozoa. Scale bar, 50 μm. (**F**) Spermatozoa from the *Ttc6^−/−^* mice can fertilize normal mouse oocytes using in vitro fertilization. Morphology of two-pronucleus, two-cell stage, and blastula-stage embryos. Scale bar, 100 μm. (**G**) The fertilization capacity of *Ttc6^−/−^* sperm with oocytes in vitro was reduced, yet the fertilized eggs were still able to develop to the blastula stage, showing no significant difference from those from *Ttc6^+/+^* sperm (*n* = 3). Data are presented as the mean ± SEM. Two-tailed Student’s *t* test. * *p* < 0.05. ns: no significance.

The subfertility observed in *Ttc6^−/−^* male mice might result from the faulty maturation of spermatozoa and/or from impaired fertilization processes. We characterized sections of the epididymis in *Ttc6^−/−^* mice using hematoxylin staining and found no discernible difference in sperm count in the *Ttc6^−/−^* caudal epididymis compared with that of the *Ttc6^+/+^* mice (Figure 3A,B). Further immunofluorescence examination using antibodies specific to the flagellar protein tubulin and its glycation revealed no anomalies in the formation of the flagellum in *Ttc6^−/−^* testes (Figure 3C). Thus, we concluded that the disruption of *Ttc6* exerts a minimal impact on spermatogenesis.

### 3.4. Fertility Impairment in Ttc6^−/−^ Male Mice Is Linked to Faulty Sperm–Egg Interaction and Zona Pellucida Binding

In an effort to investigate the factors underlying the observed subfertility in *Ttc6^−/−^* male mice, we extracted fertilized oocytes from superovulated wild-type females mated with either *Ttc6^+/+^* or *Ttc6^−/−^* males. While a substantial number of both the two-cell and blastula-stage embryos were recorded in the *Ttc6^+/+^* group, these embryo types were markedly scarce in the *Ttc6^−/−^* group (Figure 3D). This led us to conclude that an impediment in fertilization was the cause of the subfertility in the *Ttc6^−/−^* male mice. Consequently, we explored the possibility of compromised in vitro sperm–egg interactions or diminished fertilization efficacy of *Ttc6^−/−^* sperm. Notably, *Ttc6^−/−^* sperm exhibited inferior binding affinity to the zona pellucida compared with *Ttc6^+/+^* sperm when exposed to cumulus-free eggs (Figure 3E). Although the fertilizing capacity of *Ttc6^−/−^* sperm with oocytes in vitro was inhibited, the resultant embryos managed to progress to the blastula stage, displaying no considerable discrepancy with those derived from *Ttc6^+/+^* sperm (Figure 3F,G). These findings imply that the binding ability of sperm to the zona pellucida might not be an essential determinant of sperm fertility.

### 3.5. Knockout of Ttc6 Affects Sperm Motility 

**Figure 4:**
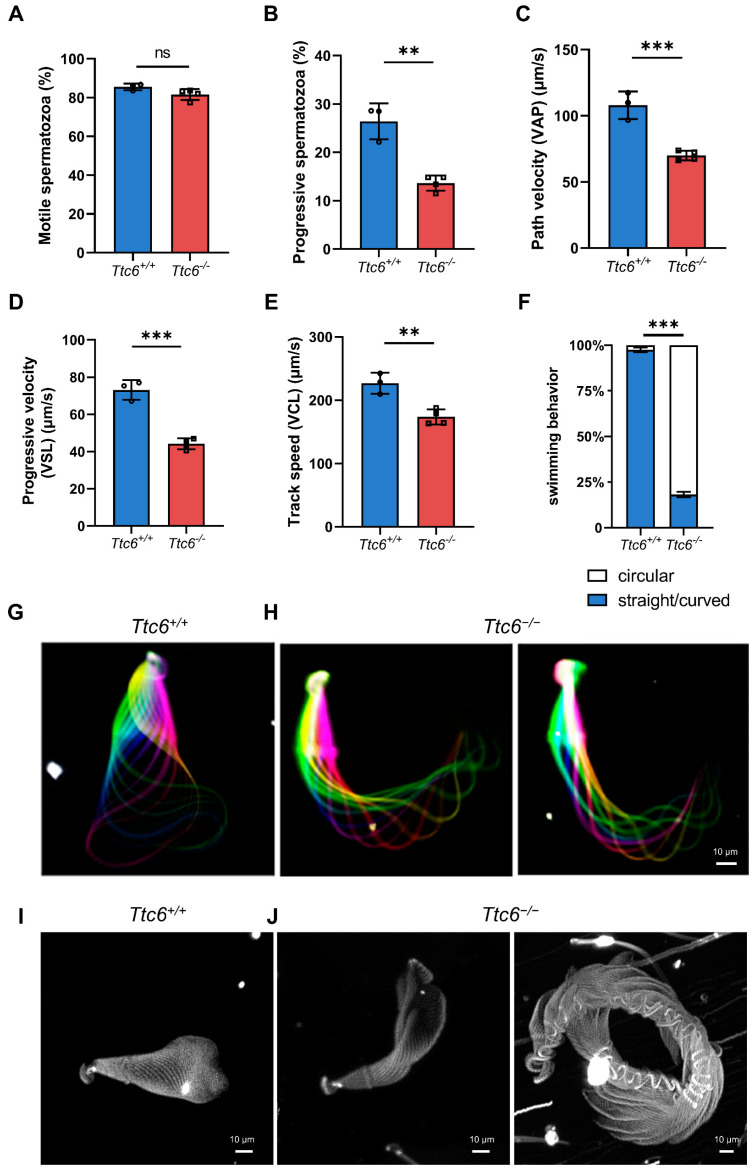
Sperm motility is altered in *Ttc6^−/−^* mice. (**A**) The frequency of motile spermatozoa in *Ttc6^−/−^* mice (*n* = 3) did not differ from that of *Ttc6^+/+^* mice (*n* = 4). Data are presented as the mean ± SEM. Two-tailed Student’s *t*-test; ns: no significance. (**B**) The frequency of progressive spermatozoa in *Ttc6^−/−^* mice (*n* = 3) was decreased compared with that of *Ttc6^+/+^* mice (*n* = 4). Data are presented as the mean ± SEM. Two-tailed Student’s *t*-test; ** *p* < 0.01. (**C**) The average path velocity (VAP) of spermatozoa from *Ttc6^+/+^* (*n* = 3) and *Ttc6^−/−^* mice (*n* = 4). Data are presented as the mean ± SEM. Two-tailed Student’s *t*-test; *** *p* < 0.001. (**D**) The progressive velocity (VSL) of spermatozoa from *Ttc6^+/+^* mice (*n* = 3) and *Ttc6^−/−^* mice (*n* = 4). Data are presented as the mean ± SEM. Two-tailed Student’s *t*-test; *** *p* < 0.001. (**E**) The track speed (VCL) of spermatozoa from *Ttc6^+/+^* mice (*n* = 3) and *Ttc6^−/−^* mice (*n* = 4). Data are presented as the mean ± SEM. Two-tailed Student’s *t*-test; ** *p* < 0.01. (**G**,**H**) Color-coded time projections of representative dark-field recordings of freely swimming *Ttc6^+/+^* and *Ttc6^−/−^* sperm near the glass surface. Scale bar, 10 μm. (**I**,**J**) The swimming paths of *Ttc6^+/+^* and *Ttc6^−/−^* spermatozoa. *Ttc6^+/+^* spermatozoa swam along a twisted ribbon-like path, whereas *Ttc6^−/−^* spermatozoa swam along a circular path. Scale bar, 10 μm. (**F**) Quantification of the swimming patterns observed in (**I**,**J**). Data are presented as the mean ± SEM. Two-tailed Student’s *t*-test; *** *p* < 0.001.

Next, we released spermatozoa from the cauda and evaluated their motility following capacitation via incubation in tubal fluid. As visualized with a computer-assisted sperm analyzer, the percentage of motile sperm was not significantly different between *Ttc6^+/+^* and *Ttc6^−/−^* mice (Figure 4A). However, in *Ttc6^−/−^* male mice, there was a notable decline in the proportions of progressive sperm (26.41 ± 2.14% vs. 13.64 ± 0.79%; Figure 4B). Further analysis of the sperm motility parameters that determine the speed of sperm motion was conducted in both *Ttc6^−/−^* and *Ttc6^+/+^* mice. Declines were detected in four kinematic parameters measuring sperm velocity, including average path velocity (VAP, representing the average velocity of the sperm head through its average trajectory) (107.98 ± 6.02 mm/s vs. 69.95 ± 1.83 mm/s; Figure 4C), straight line velocity (VSL, representing the mean speed of the sperm head along a straight path connecting the initial and final positions) (73.13 ± 3.05 mm/s vs. 44.18 ± 1.46 mm/s; Figure 4D), and curvilinear velocity (VCL, representing the mean speed of the sperm head along its actual path) (226.86 ± 9.61 mm/s vs. 173.51 ± 5.97 mm/s; Figure 4E). These results strongly suggest that the reduced fertilization potential of *Ttc6^−/−^* sperm due to reduced sperm motility (asthenozoospermia) is a prevalent factor contributing to male subfertility in our mouse model.

### 3.6. Knockout of Ttc6 Alters Sperm Flagellar Beat Patterns and Causes Anomalous Sperm Swim Paths

After noting the diminished motility in *Ttc6^−/−^* sperm, our investigation continued into the specific aspects of these spermatozoa’s flagellar beating. We executed a two-dimensional examination of individual sperm cells, affixing their heads to a glass surface, consistent with the technique employed in a previous study [30]. Our analysis demonstrated that *Ttc6^+/+^* spermatozoa exhibited a characteristic symmetric flagellar beat (Figure 4G), while *Ttc6^−/−^* spermatozoa exhibited anomalously stiff flagella, which maintained a constant curvature from the middle piece to the proximal region of the principal piece (Figure 4H). This suggested that knockout of *Ttc6* altered the flagellar beat patterns of the sperm.

Symmetric flagellar beating is a prerequisite for progressive swimming, and this was missing in *Ttc6^−/−^* sperm. Next, we assessed the swimming patterns of *Ttc6^+/+^* and *Ttc6^−/−^* spermatozoa. The head tracing of free-swimming spermatozoa revealed that *Ttc6^+/+^* sperm predominantly swam along either straight or curved paths (Figure 4I). Conversely, while *Ttc6^−/−^* spermatozoa were able to swim, their movement was mostly circular (Figure 4F,J). The main deficiency in *Ttc6^−/−^* sperm cells was their nonlinear swimming pattern, a factor that evidently obstructed the sperms’ capacity to navigate to the fertilization site. Taken together, these results show that the knockout of *Ttc6* results in the alteration in sperm flagellar beat patterns and atypical sperm swimming paths, thus providing a credible explanation for the subfertility observed in *Ttc6^−/−^* males.

### 3.7. Spermatozoa from Ttc6^−/−^ Mice Exhibit Abnormal Morphology

**Figure 5:**
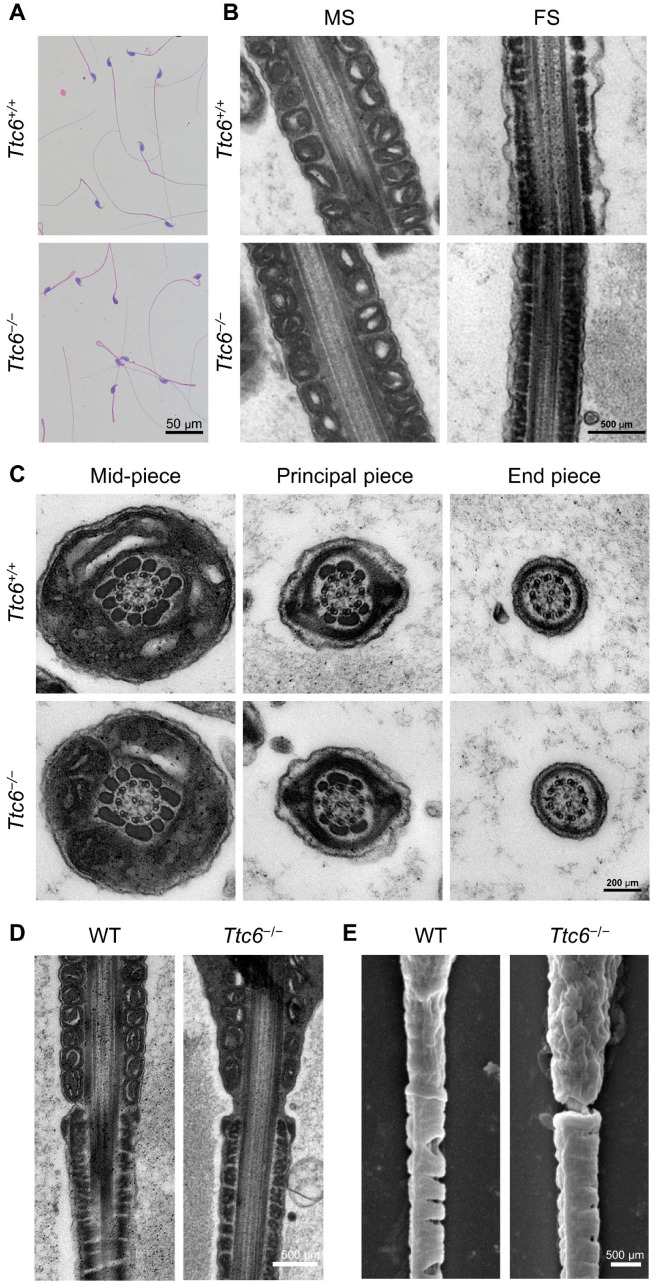
The sperm flagella in *Ttc6^−/−^* mice are abnormal. (**A**) Morphology of the cauda epididymal spermatozoa from *Ttc6^+/+^* and *Ttc6^−/−^* mice. Scale bar, 50 μm. (**B**) Representative TEM images of the sperm mitochondrial sheath (MS) and fibrous sheath (FS) from *Ttc6^+/+^* and *Ttc6^−/−^* mice. Scale bar, 500 μm. (**C**) Representative TEM images showing cross-sections of the midpiece, principal piece, and end piece of sperm flagella from *Ttc6^+/+^* and *Ttc6^−/−^* mice. Scale bar, 200 μm. (**D**) Representative TEM images showing the unsheathed flagellar region at the junction between the midpiece and principal piece. Scale bar, 500 μm. (**E**) Representative scanning electron microscope images showing the unsheathed flagellar region at the junction between the midpiece and principal piece. Scale bar, 500 μm.

The motility of sperm has been shown to be associated with their structural integrity and supplemental energy [1,31]. To test whether the diminished motility of *Ttc6^−/−^* spermatozoa is attributable to defects in the sperm flagellum structure, we performed morphological analysis of spermatozoa from the epididymis. This analysis revealed that *Ttc6^−/−^* spermatozoa had a hairpin phenotype, which is characterized by a sharp bend at the junction between the flagellum midpiece and principal piece (Figure 5A). We proceeded to investigate the ultrastructure of the sperm flagellum in the cauda epididymis using transmission electron microscopy (TEM). The TEM images revealed that *Ttc6^−/−^* spermatozoa still had a normal structural organization in both the mitochondrial sheath and fibrous sheath (Figure 5B). Further exploration of the flagellar ultrastructure showed that *Ttc6^−/−^* spermatozoa did not display abnormalities in the 9 + 2 axonemal structure compared with *Ttc6^+/+^* spermatozoa (Figure 5C). However, TEM and scanning electron microscope analyses of longitudinal sections of *Ttc6^−/−^* sperm flagella showed an unsheathed flagellar region at the junction between the midpiece and principal piece (Figure 5D,E), which was consistent with the hairpin phenotype identified in the morphological analysis of the spermatozoa. Collectively, these results indicate that reduced sperm motility in *Ttc6^−/−^* mice is attributable to structural anomalies in the flagellum.

## 4. Discussion

Previous research has indicated that proteins from the TRP family, including *Ttc21a* and *Ttc29*, are required for the proper development and functioning of spermatozoa and thus play an essential role in male fertility [25,26]. In our current study, we discovered that TTC6, a protein associated with the sperm flagellum, is predominantly expressed in the testes and that knockout of *Ttc6* in mice leads to a significant reduction in sperm motility and a change in sperm swimming direction, causing male subfertility. Additionally, we found that the decrease in motility of *Ttc6^−/−^* sperm is due to an abnormal flagellar structure. Knockout of *Ttc6* caused a sharp bend at the junction of the midpiece and principal piece, and super-resolution imaging revealed an unsheathed region of the flagella at this junction. These findings suggest that TTC6 is essential for maintaining the structural integrity of the flagellum annulus region, which in turn supports sperm motility and direction.

We found that *Ttc6* is predominantly expressed in the testes of mice, starting at postnatal day 21, and continuously thereafter in adult mice. This pattern aligns with the appearance of postmeiotic round spermatids and the initial formation of flagella [32,33]. In early round spermatids, the genesis of sperm flagella initiates as the axoneme elongates from the distal centriole [31]. Within the context of the tetratricopeptide repeat protein family, TTC6 emerges as a significant member and has been identified as a plausible candidate gene for ciliopathies, stemming from contemporary investigations into the pathological genome of such conditions [29]. The construction of the mammalian sperm flagellum embodies an axoneme featuring a 9 + 2 microtubule pattern, akin to the structural organization found in motile cilia [33]. Our conducted research has elucidated that in the absence of TTC6, as observed in *Ttc6^−/−^* mice (Figure 5A,D,F), there is a discernible hindrance in the formation of axonemes during early round spermatids. This finding underscores the indispensable role of TTC6 in the biological process of sperm flagellum assembly.

For mammals, an essential condition for successful fertilization is the ability of sperm to navigate through the female reproductive tract. This mobility enables the sperm to locate and fertilize the ovum [34,35]. Testicular sperm are typically immotile, and, to participate in fertilization, mouse spermatozoa must undergo progressive functional maturation in the epididymis [7,8]. As spermatozoa transition from the caput epididymis, their movement is initially slow and irregular. However, upon moving through the corpus epididymis, there is a sharp increase in motility, which continues as they traverse the cauda epididymis and the vas deferens [36]. Sperm motility is one of the key factors determining fertility [37]. An assessment of *Ttc6^−/−^* sperm movement, conducted through an automated CASA system, has pinpointed an issue with motility. Further scrutiny with the aid of high-speed cameras exposed an irregularity in the flagellar waveform, which frequently retained an antihook shape. This unusual conformation contributed to a noticeable decrease in the swimming velocity of the mutant sperm.

A previous study has proven that TEKT4 is vital for ensuring the correct synchronized beating of the sperm flagellum, a crucial aspect of male reproductive physiology. In instances where TEKT4 is null, sperm display a marked decrease in forward progressive velocity and a disorganized propagation of waveform along the flagellum [20]. In the absence of TCTE1, male mice suffer from sterility, a condition attributed to asthenozoospermia. CASA shows that this reduced motility originates from a dysfunction in flagellar performance, leading to sperm tails bending to one side, thus forming a circular path for sperm [21]. In *Cfap97d1*-knockout mice, the spermatozoa exhibited irregular motility, a behavior marked by common stalling at the antihook position. This distinct pattern was correlated with the loss of *Cfap97d1*, leading to a unique phenotype. This phenotype was associated with heterogeneity in the axonemal doublets, often coinciding with the loss of the fourth doublet in sperm retained within the epididymis [22]. Recent research has shown that tubulin glycation influences the waveform of sperm flagella and regulates their swimming direction. An absence of tubulin glycation has been linked to abnormal dynein arm configurations within sperm axonemes, presumably leading to a shift in the force-generation profile and an aberrant beating pattern [23]. The underlying method of controlling the beat of the flagellum continues to be an area requiring further exploration.

The sperm annulus, which forms at the sperm neck during spermatid elongation, later migrates along the axoneme to the junction of the midprincipal piece in spermatozoa [7,8]. Electron microscopy analysis of the sperm annulus revealed an electron-dense ring structure between the midpiece and principal piece of all mammalian spermatozoa. High-resolution imaging showed that this annulus/ring structure consisted of densely packed filamentous structures. Considering that the sperm flagella were unsheathed at the junction between the midpiece and principal piece in *Ttc6^−/−^* spermatozoa, we inferred that *Ttc6* is required for maintaining the structural integrity of the annulus. The sperm annulus has been the subject of morphological studies for many years, with ongoing investigations into its composition and multifaceted functions. Research has elucidated three primary roles played by the sperm annulus ring: firstly, it provides structural and mechanical backing; secondly, it operates as a selective barrier to confine specific proteins to designated membrane domains; and, thirdly, it governs the distribution of mitochondria [7,9,34,35,36]. Within the context of our investigation, we observed a distinct rotating pattern in the motion track of sperm, accompanied by a conspicuous abnormal bright spot track (Figure 4H) in proximity to the annulus. Interestingly, we first noted unsheathed flagella at the midpiece and principal piece junction in the *Ttc6^−/−^* corpus epididymis. It is plausible that the absence of TTC6 may lead to a destabilized annulus in spermatozoa, resulting in failures in structural and mechanical support. This instability could be linked to the decreased motility observed in the corpus epididymis. 

Along with increased motility, spermatozoa undergo transformations in nuclear compaction, plasma membrane composition, cytoskeletal structure, protein payload, and noncoding RNA payload within the epididymis [38]. Additionally, the mammalian epididymis offers a unique intraluminal environment for the morphological and biochemical changes that take place in mature spermatozoa [39]. Consequently, TTC6 might play an essential role in addressing challenges related to prolonged storage and varying environmental conditions in the epididymis, ultimately helping to maintain the axonemal integrity of the sperm flagellum. Despite the impaired ability of *Ttc6^−/−^* sperm to bind effectively to the zona pellucida, our in vitro fertilization assays showed that this deficiency does not necessarily compromise their fertilization capacity. This conclusion aligns with numerous prior studies that have demonstrated a disconnect between zona pellucida binding ability and successful fertilization both in vitro and in vivo [40,41,42,43,44]. Our research thus promises to enhance genetic diagnostic methods and could potentially inform clinicians in devising suitable therapeutic strategies.

## 5. Conclusions

In summary, this study reveals new insights into the unexplored function of the *Ttc6* gene and illustrates that *Ttc6* is vital in preserving the structural integrity of the flagellum and thereby controlling sperm motility and fertilization properties in mice.

## Data Availability

All data are included in the manuscript.

## Supplementary Materials

The following supporting information can be downloaded at: https://www.mdpi.com/article/10.3390/cells12162091/s1, Figure S1: *Ttc6* knockout leads to male subfertility but unaltered hormone levels; Figure S2: Disruption of *Ttc6* had no effect on acrosome biogenesis.
