# Supplementary material for: TTC6-Mediated Stabilization of the Flagellum Annulus Ensures the Rapid and Directed Motion of Sperm
Source: Cells. 2023 Aug 18;12(16):2091. doi: 10.3390/cells12162091 (PMC10453820; doi:10.3390/cells12162091)
Supplement: Supplementary file 1 [file cells-12-02091-s001.zip › cells-2538834-SI.pdf]

TTC6-mediated stabilization of the flagellum annulus ensures the rapid and directed motion of sperm

Supplemental Figures

A

genotype	male #	litters born	litter size1	litter size2	litter size3	litter size4	litter size5	litter size6
+/+	1	6	7	8	8	9	7	7
	2	6	8	9	5	4	6	6
	3	6	10	6	5	8	7	7
	4	6	8	8	3	10	9	8
	5	6	7	6	8	5	7	7
	6	6	5	7	10	8	6	9
−/−	1	1	2					
	2	1	1					
	3	0						
	4	0						
	5	0						
	6	0						

B

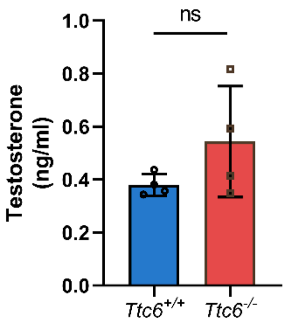

Supplementary Figure S1. *Ttc6* knockout leads to male subfertility but unaltered hormone levels. (**A**) The mating test using wild type female mice coupled with *Ttc6*$^{+/+}$ or *Ttc6*$^{-/-}$ adult male mice. The number of deliveries for each *Ttc6*$^{+/+}$ or *Ttc6*$^{-/-}$ adult male mouse is described. Litter size is the number of pups in each delivery. (**B**) Hormone levels remained unaltered in *Ttc6*$^{-/-}$ mice. Estrone, cortisol, progesterone, and testosterone levels were compared between *Ttc6*$^{+/+}$ and *Ttc6*$^{-/-}$ mice. Data are presented as the mean ± SEM. Two-tailed Student's t-test; ns: no significance.

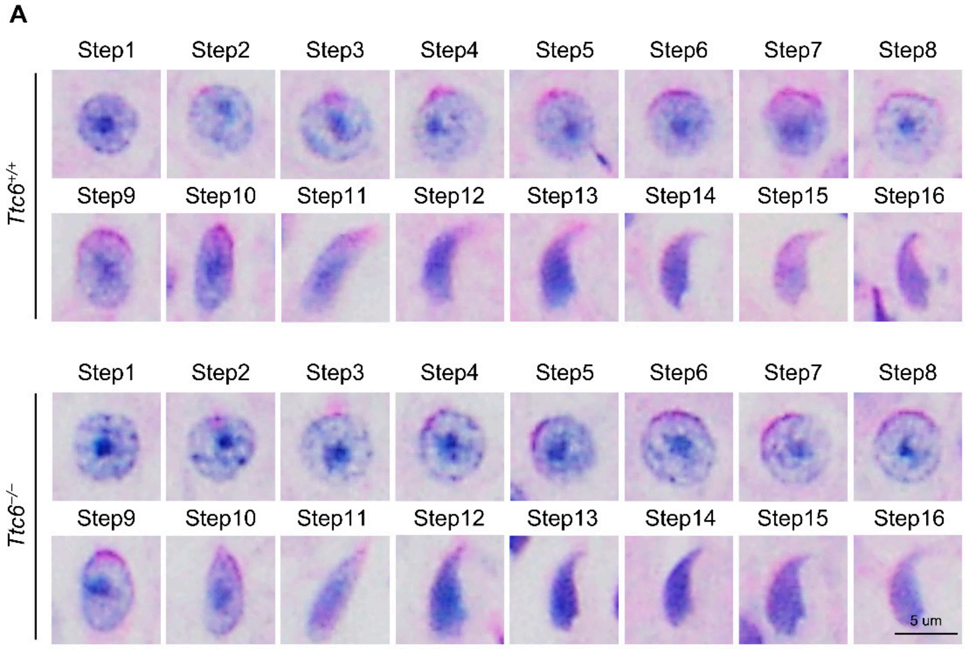

Supplementary Figure S2. Disruption of *Ttc6* had no effect on acrosome biogenesis. (**A**) Step 1 through Step 16 of spermatogenesis in *Ttc6*$^{+/+}$ and *Ttc6*$^{-/-}$ testis stained with PAS-hematoxylin. Scale bar, 5 μm.
